# Recent Advances of MEMS Resonators for Lorentz Force Based Magnetic Field Sensors: Design, Applications and Challenges

**DOI:** 10.3390/s16091359

**Published:** 2016-08-24

**Authors:** Agustín Leobardo Herrera-May, Juan Carlos Soler-Balcazar, Héctor Vázquez-Leal, Jaime Martínez-Castillo, Marco Osvaldo Vigueras-Zuñiga, Luz Antonio Aguilera-Cortés

**Affiliations:** 1 Micro and Nanotechnology Research Center, Universidad Veracruzana, Calzada Ruiz Cortines 455, Boca del Río, Veracruz 94294, Mexico; jaimartinez@uv.mx; 2 Engineering Faculty, Universidad Veracruzana, Calzada Ruiz Cortines 455, Boca del Río, Veracruz 94294, Mexico; jsoler@uv.mx (J.C.S.-B.); mvigueras@uv.mx (M.O.V.-Z.); 3 Electronic Instrumentation Faculty, Universidad Veracruzana, Cto. Gonzálo Aguirre Beltran S/N, Xalapa, Veracruz 91000, Mexico; hvazquez@uv.mx; 4 Departamento de Ingeniería Mecánica, DICIS, Universidad de Guanajuato, Carr. Salamanca-Valle de Santiago km 3.5+1.8 km, Palo Blanco, Salamanca, Guanajuato 36885, Mexico; aguilera@ugto.mx

**Keywords:** Lorentz force, magnetic field sensor, MEMS, resonators, sensing technique

## Abstract

Microelectromechanical systems (MEMS) resonators have allowed the development of magnetic field sensors with potential applications such as biomedicine, automotive industry, navigation systems, space satellites, telecommunications and non-destructive testing. We present a review of recent magnetic field sensors based on MEMS resonators, which operate with Lorentz force. These sensors have a compact structure, wide measurement range, low energy consumption, high sensitivity and suitable performance. The design methodology, simulation tools, damping sources, sensing techniques and future applications of magnetic field sensors are discussed. The design process is fundamental in achieving correct selection of the operation principle, sensing technique, materials, fabrication process and readout systems of the sensors. In addition, the description of the main sensing systems and challenges of the MEMS sensors are discussed. To develop the best devices, researches of their mechanical reliability, vacuum packaging, design optimization and temperature compensation circuits are needed. Future applications will require multifunctional sensors for monitoring several physical parameters (e.g., magnetic field, acceleration, angular ratio, humidity, temperature and gases).

## 1. Introduction

Microelectromechanical systems (MEMS) allow the development of devices that are composed by mechanical and electrical components with a feature size in the micrometer-scale. MEMS devices include signal acquisition and processing, actuators and control mechanisms [1]. These devices provide the following advantages: small size, low power consumption, high sensitivity and reduced fabrication cost [2]. Recently, several researchers [3,4,5,6,7,8] have designed MEMS magnetic field sensors for potential applications such as biomedical, telecommunications, navigation and non-destructive testing. Most of these sensors include resonators that operate with the Lorentz force, which is generated by the interaction between an external magnetic field and an electrical current. It causes deformations of the resonators, which are increased at resonance. These deformations can be measured using capacitive, piezoresistive and optical sensing techniques. Thus, MEMS sensors can have better resolution than Hall effect and search coil sensors [9]. On the other hand, a superconducting quantum interference device (SQUID) is expensive and requires a sophisticated infrastructure [10]. Other conventional devices include the anisotropic magnetoresistive (AMR) and fluxgate sensors. Auto-calibration systems are necessary for AMR sensors, which are saturated with small magnetic fields (close to few mT) [11,12]. Otherwise, fluxgate sensors need a complex fabrication of their magnetic core and coils [13]. 

MEMS magnetic field sensors use a bias source and a signal processing system. For instance, Dominguez-Nicolás et al. [4] designed a signal conditioning system, implemented in a printed circuit board (PCB), for a MEMS magnetic field sensor. It obtains the sensor response in voltage or current mode for a magnetic field range from −150 μT to +150 μT. The sensor employs a virtual instrument design through Labview software to visualize the 4–20 mA output signal. For industrial applications, the sensor response is processed with a data acquisition card and a programmable logic controller (PLC). Thus, MEMS sensors could commercially compete against conventional magnetic field sensors in a wide variety of applications.

## 2. Design and Fabrication

Design stage is very important in determining the suitable structural configuration, materials, operation principle and sensing technique of MEMS magnetic field sensors. In this stage, MEMS designers can use analytical and numerical methods to predict the performance of MEMS sensors. Designers must consider the rules of the fabrication process to avoid mistakes that affect the performance of the sensors. To obtain reliable designs, designers must have a high level of fabrication and packaging knowledge. In the design stage, simulation and modeling tools are necessary to predict the behavior of MEMS sensors. These tools assist designers in selecting the best fabrication process and materials for the sensors. To develop commercialized MEMS sensors, designers must satisfy the following criteria: (1) optimal design; (2) packaging design; (3) reliable materials properties and standard fabrication process; (4) suitable design and simulation tools; (5) reduction of electronic noise and parasitic capacitances; (6) reliable signal processing systems and; (7) reliability testing.

**Figure 1:**
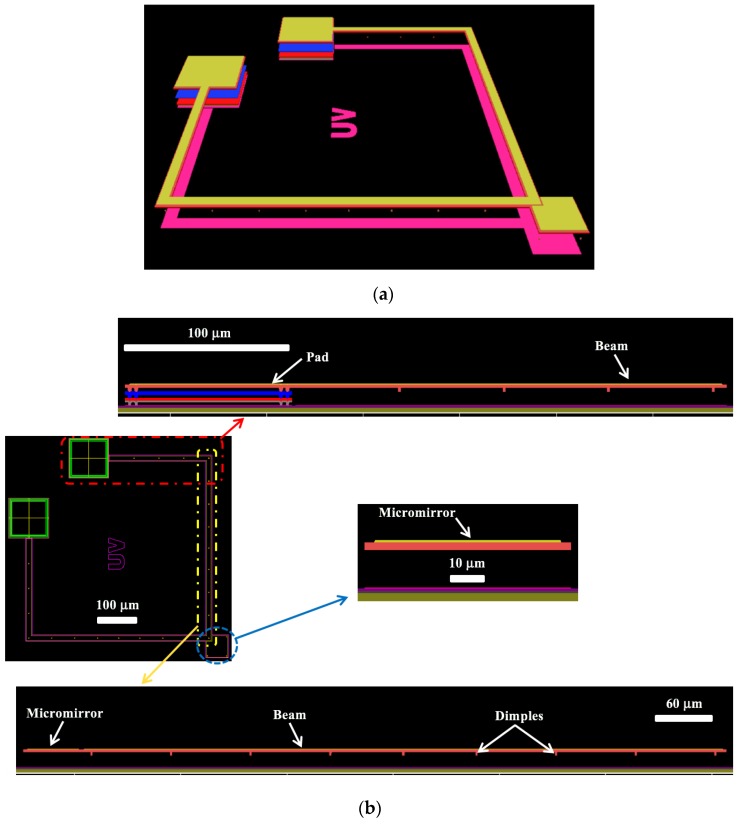
Layout of a magnetic field sensor with a polysilicon resonator, which is based on SUMMiT V process: (**a**) 3D; and (**b**) 2D view of the main mechanical components [19]. Reprinted with permission from [19]. Copyright©2013, Bentham Science Publishers.

The selection of the sensors fabrication process must consider several factors such as materials, operation principle, dimensions, operating environmental conditions, signal conditioning processes and sensing techniques [14]. The design rules of each fabrication process must be considered during the sensors design stage. For this design, several MEMS design tools can be used including MEMSCAP^TM^ [15], coventorWare^TM^ [16], IntelliSuite^TM^ [17] and Sandia Ultra-planar Multi-level MEMS Technology (SUMMiT V) [18]. These design tools have modules to create the sensor layout and check the design rules, as well as simulating the steps of the micromachining process. These advantages may reduce the work time related to the sensor’s design. In addition, the suitable design of a sensor depends on the designer’s experience in using efficient methodology processes in the selection of better materials and micromachining processes. Figure 1 depicts the layout of a magnetic field sensor based on the SUMMiT V process [19].

Designers could employ the following methodology to design and fabricate MEMS magnetic field sensors:

(1) Application specification. In this stage, designers must know all the technical information of the sensor application such as magnetic field range, environmental conditions, response time and resolution.
(2) Conceptual design. This stage includes several conceptual designs of magnetic field sensors to satisfy the requirements of the applications. Next, these designs are evaluated to select the best conceptual design.
(3) Detailed design. Analytical and numerical methods are used to predict the sensor’s performance. These designs must incorporate the sensor packaging, which depends of different factors such as materials, operation pressure, cost, sensing technique and environmental conditions. High temperatures can be generated during the sensor packaging, affecting its behavior. For this, element finite method (FEM) models can be used to estimate the packaging temperature effect on the sensor behavior.
(4) Fabrication process. Designers identify the sensor requirements and characteristics of potential fabrication processes. Next, the designer selects the best fabrication process or develops a new process, in which must consider the materials, etching steps, design rules, technical limitations and cost.
(5) Material properties verification. Test structures can be fabricated on the same sensor wafer to measure several materials properties of the sensors such as Young’s modulus, facture strength, thermal conductivity, electrical resistivity and dielectric constant.

Generally, the fabrication of MEMS magnetic field sensors can be realized using bulk or surface micromachining processes. These processes use silicon as their main material due to its important mechanical and electrical properties. For instance, silicon has minimum mechanical hysteresis and large rupture stress close to 1 GPa. In addition, silicon doped with phosphorus or boron can improve its electrical properties. 

**Figure 2:**
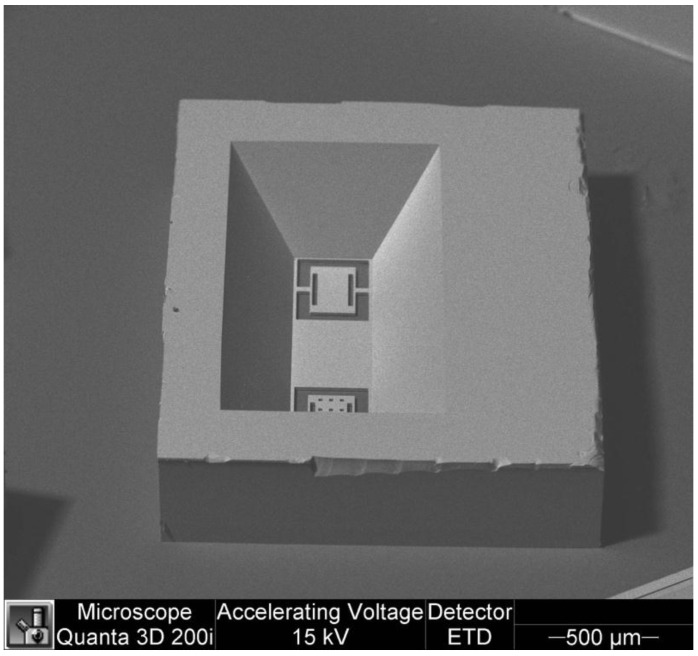
SEM image of a die reverse-side with two silicon structures which were etched using the KOH in bulk micromachining process. (Courtesy of A. L. Herrera-May, Universidad Veracruzana).

**Figure 3:**
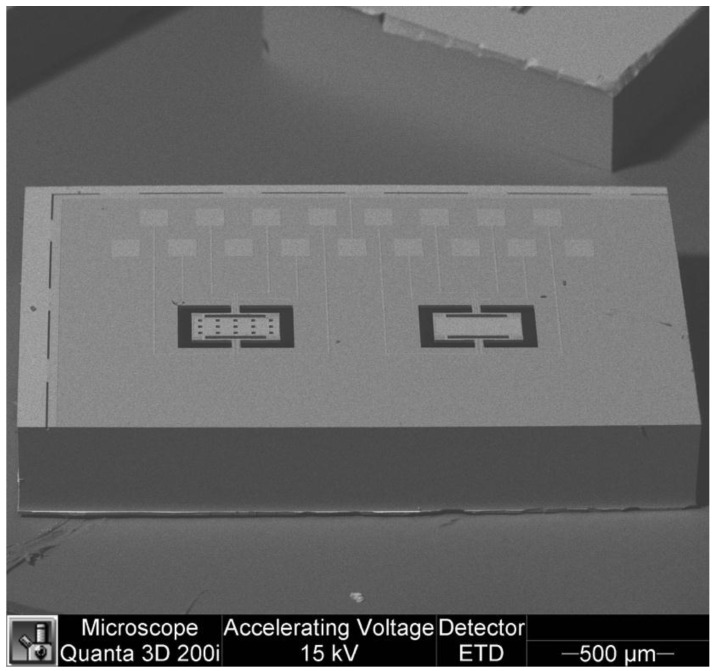
SEM image of two magnetic field sensors formed by silicon resonators, which are fabricated using a bulk micromachining process. (Courtesy of A. L. Herrera-May, Universidad Veracruzana).

The bulk micromachining process employs wet and dry etching techniques to fabricate features in materials through isotropic and anisotropic etching, respectively [20]. Wet etching is a chemical process that can reach an anisotropic directional etching in crystalline materials (e.g., silicon) although, it can get an isotropic etching in an amorphous material (e.g., silicon dioxide). For instance, potassium hydroxide (KOH) is a directional wet etchant for crystalline silicon, which etches 100 times faster on the (100) plane than on the (111) plane (see Figure 2). KOH etches very slowly the silicon nitride or silicon dioxide, which can be used as etch masks. In wet etching, the etched depth depends of several variables such as etched time, temperature, chemical agitation and solution concentration. The disadvantages of the wet etching can be overcome using dry etching (e.g., plasma etching). Dry etching has advantages such as anisotropic etch, repeatable process and it is easy to start and stop the etched process. Plasma etching uses a flux of ions, electrons, radicals and neutral particles to etch the material surface. It includes reactive ion etching (RIE), deep reactive ion etching (DRIE) and high-density plasma etching (HDP). Figure 3 shows a SEM image of two magnetic field sensors composed by silicon resonators with piezoresistive sensing, which were fabricated using a bulk micromachining process. These sensors were designed by researchers at Micro and Nanotechnology Research Center (MICRONA-UV, Boca del Río, Mexico) into collaboration with Microelectronics Institute of Barcelona (IMB-CNM, CSIC, Bellaterra, Spain).

**Figure 4:**
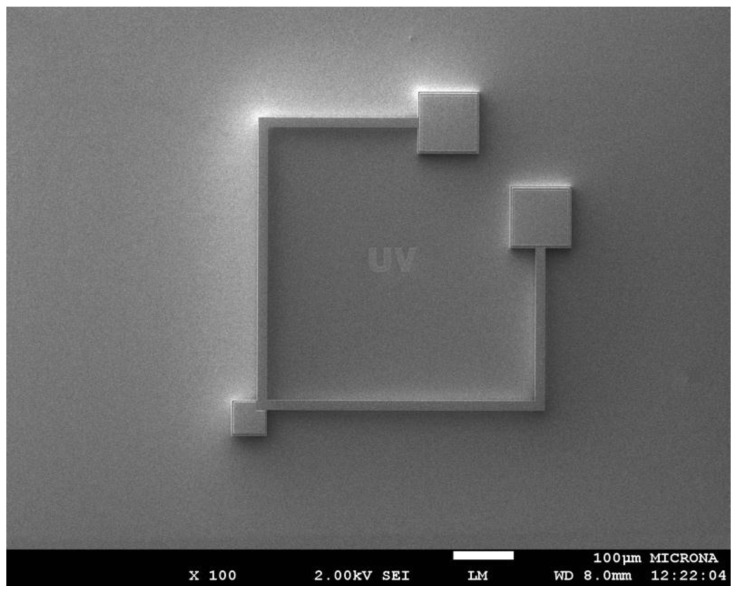
SEM image of magnetic field sensor composed by a polysilicon resonator, which is fabricated using a surface micromachining process. (Courtesy of A. L. Herrera-May, Universidad Veracruzana).

Surface micromachining process is a fabrication process of MEMS devices that uses the deposition, patterning and etching of different materials layers on a substrate [20]. Commonly, these layers are employed as structural and sacrificial layers. The sacrificial layers protect the structural layers during the etching process and define the mechanical structure of the MEMS devices. Finally, sacrificial layers are removed using specific chemical substances. Figure 4 shows a polysilicon resonator of a magnetic field sensor, which is fabricated using a surface micromachining process. It was designed by researchers at Micro and Nanotechnology Research Center (MICRONA-UV) into collaboration with Sandia National Laboratories.

Planar fabrication methods of the microelectronic industry can be used in surface micromachining process. For instance, this process employs tools of the microelectronic industry for depositing the structural and sacrificial layers, as well as the patterning and etching of layers. Thus, devices structures are obtained through etching of the structural layers, which are anchored to the substrate and others structural layers. In the deposition process of structural and sacrificial layers, residual stress gradients can be generated on the structural layers, which can affect the performance of the MEMS sensors. These stress gradients are caused by deposition conditions such as high temperature values and materials layers with different thermal dilation coefficients. The residual stress gradients can be decreased using post-deposition annealing steps.

### 2.1. Performance of MEMS Magnetic Field Sensors

**Figure 5:**
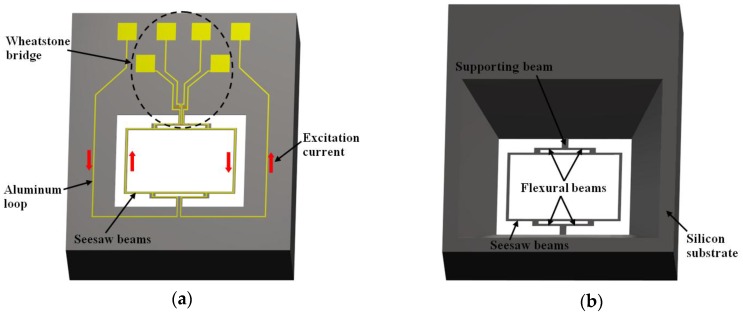
3D schematic view of the (**a**) upper and (**b**) bottom design of magnetic field sensor, which contains a silicon resonator, an aluminum loop and a Wheatstone bridge [21]. Reprinted with permission from [21]. Copyright©2011, Elsevier B.V.

**Figure 6:**
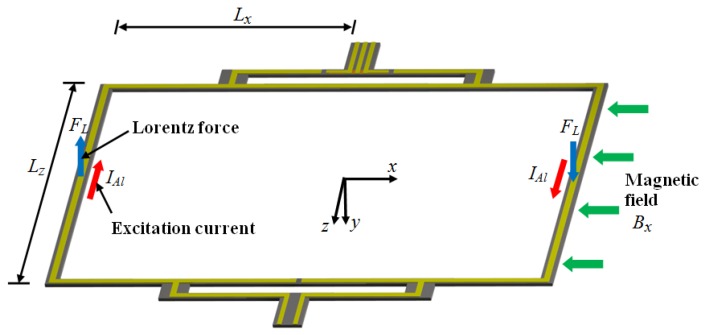
3D schematic view of the performance of a MEMS magnetic field sensor formed by a rectangular loop of silicon beams, an aluminum loop and piezoresistive sensing [21]. Reprinted with permission from [21]. Copyright©2011, Elsevier B.V.

MEMS magnetic field sensors use resonators to increase their sensitivity due to large strains of the sensor structure. The sensor structure operates at resonance through Lorentz forces or electrostatic forces whose displacements are related to the magnitude of the applied magnetic field. These displacements can be detected using piezoresistive, capacitive or optical sensing techniques. For instance, Figure 5a,b depicts the design of a magnetic field sensor which contains a resonator and piezoresistive sensing [21]. This sensor is formed by a rectangular loop of silicon beams, an aluminum loop and a Wheatstone bridge, as shown in Figure 6. This sensor exploits the Lorentz force (*F_L_*) that is generated by the interaction of the electrical current with an external magnetic field (*B_x_*) parallel to the sensor structure:

(1) $$F_{L}=I_{Al}B_{x}L_{z}$$

with

(2) $$I_{Al}=\sqrt{2}I_{RMS}\sin(\omega t)$$

where *L_z_* is width of the rectangular loop, *ω* and *I_RMS_* are circular frequency and root-mean-square (RMS) of the sinusoidal electrical current (*I_Al_*) supplied to the aluminum loop, respectively.

The sensor structure has a deflection and strain generated by the Lorentz force, causing a change (△*R_i_*) of the initial resistance (*R_i_*) of two p-type piezoresistors:

(3) $$\Delta R_{i}=\pi_{l}E\varepsilon_{l}Q_{T}R_{i}$$

where *π_l_* is longitudinal piezoresistive coefficient, *ε_l_* is longitudinal strain of the piezoresistors under static load, *E* is Young’s modulus of the piezoresistor material and *Q_T_* is total quality factor of the resonant structure.

The output voltage (*V_o_*) of the Wheatstone bridge changes due to variation of the piezoresistors resistance:

(4) $$V_{o}=\frac{1}{2}\pi_{l}E\varepsilon_{l}R_{i}V_{i}$$

where *V_i_* is input voltage applied to the Wheatstone bridge.

The sensor sensitivity (*S*) is obtained as the ratio between the output voltage (Δ*V_o_*) of the Wheatstone bridge to the magnetic field shift (Δ*B_x_*):

(5) $$S=\frac{\Delta V_{o}}{\Delta B_{x}}$$

### 2.2. Quality Factor

The quality factor of a resonator affects its sensitivity and resolution. This factor can be determined as the ratio of the total energy stored in resonator (*E_s_*) to the energy lost per cycle (*E_C_*) caused by the damping sources:

(6) $$Q=\frac{2\pi E_{s}}{E_{c}}$$

For small displacements of the resonator, the quality factor is related with the damping ratio (*ζ*):

(7) $$Q=\frac{1}{2\zeta }$$

The main damping sources of MEMS resonators are the following: fluid damping, support damping and thermoelastic damping. Fluid damping is due to energy loss to a surrounding fluid and its value is affected by parameters such as the viscosity and pressure of the fluid, resonator size, vibration mode and separation of the resonator with respect to the adjacent surfaces [22]. This damping decreases when the fluid pressure is reduced to vacuum, which increases the resonator motions, the sensitivity and resolution of the sensor. Therefore, a vacuum packaging can improve the performance of magnetic field sensors. The fluid damping is the most dominant source of energy dissipation for resonators operating at ambient pressure. 

Support damping is generated by the vibration energy dissipation in the supports of resonators. These supports absorb part of the vibration energy of a resonator, which depends of the support type and dimensions, as well as the vibration mode of the sensor structure [23].

Thermoelastic damping of a resonator is due to the oscillating temperature gradient generated during the resonator vibration [24]. This temperature gradient causes thermal energy loss that can be a dominant damping when the resonator operates at vacuum pressure. 

Total quality factor (*Q_T_*) of a resonator can be determined considering different damping sources.

(8) $$\frac{1}{Q_{T}}=\frac{1}{Q_{f}}+\frac{1}{Q_{s}}+\frac{1}{Q_{t}}$$

where *Q_f_*, *Q_s_*, and *Q_t_* are quality factors associated with the fluid damping, support damping and thermoelastic damping, respectively.

### 2.3. Sensing Techniques

MEMS magnetic field sensors can employ different sensing techniques such as capacitive, optical, or piezoresistive. These techniques allow the conversion of magnetic field signals into electrical or optical signals, respectively. In addition, MEMS sensors need signal conditioning systems with low electronic noise and parasitic capacitances. In the following paragraphs, several examples of MEMS magnetic field sensors with different sensing techniques are discussed.

**Figure 7:**
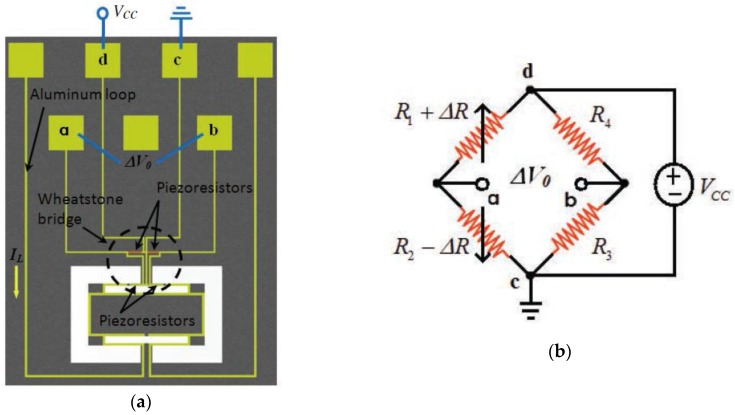
(**a**) Schematic view of a MEMS magnetic field sensor with (**b**) piezoresistive sensing through a Wheatstone bridge composed by four p-type piezoresistors [25]. Reprinted with permission from [25]. Copyright©2015, IOP Publishing.

Figure 7 shows the schematic view of the main components of a MEMS magnetic field sensor, which uses a piezoresistive sensing through a Wheatstone bridge with four p-type piezoresistors [25]. The sensor structure is formed with a silicon plate (400 × 150 × 15 μm^3^) supported by four bending beams (130 × 12 × 15 μm^3^). The plate oscillates due to the interaction between the sinusoidal electrical current and magnetic field parallel to the plate. This oscillation generates a bending motion (136.52 kHz) of the beams, which contain two piezoresistors (40 × 8 × 1 μm^3^). It alters the resistance of two piezoresistors, modifying the output voltage of the Wheatstone bridge. Thus, the sensor has an electrical output response for monitoring magnetic fields at atmospheric pressure. This sensor has a quality factor of 842 at atmospheric pressure, sensitivity of 403 mV·T^−1^, theoretical resolution of 143 nT·Hz^−1/2^, theoretical noise of 57.5 nV·Hz^−1/2^ and power consumption close to 10 mW. These technical parameters of the MEMS sensor are adequate for monitoring residual magnetic fields into ferromagnetic tubes. However, this sensor registered a voltage offset and a non-lineal electrical response.

**Figure 8:**
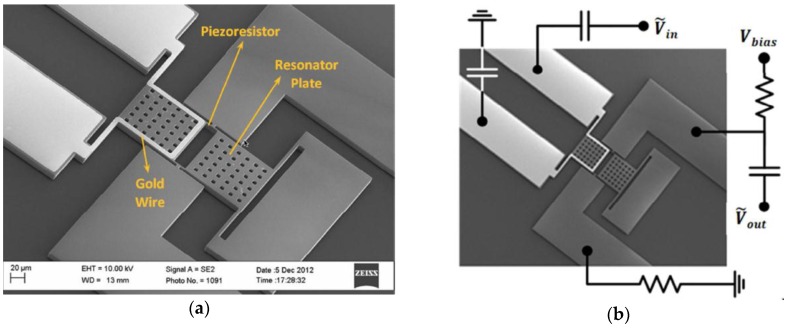
SEM image of the (**a**) main elements of a MEMS magnetic field sensor with piezoresistive readout, which is composed by a dual-plate silicon resonator with a gold trace and piezoresistors; (**b**) Electrical connections of the magnetic field sensor [26]. Reprinted with permission from [26]. Copyright©2014, IEEE.

Mehdizadeh et al. [26] designed a MEMS magnetic field sensor formed by a dual-plate silicon resonator (10 μm thickness) with a gold trace (10 μm width and 200 nm thickness) on one of its two plates (see Figure 8). Two narrow beams in the middle of the resonator connecting the two silicon plates have behave as piezoresistors. It is due to the periodic tensile and compressive stress when the resonator oscillates in-plane vibration mode. This Lorentz force-based sensor is fabricated using a low-resistivity n-type silicon-on-insulator (SOI) substrate. The quality factor of this resonator has amplification from 1140 to 16,900 at atmospheric pressure. This sensor improves its sensitivity by increasing the resonator vibration amplitude. The sensor has a sensitivity of 262 mV·T^−1^ in air, a resonant frequency of 2.6 MHz and a quality factor of 16,900. Nevertheless, the MEMS sensor requires more studies of the effects of temperature on its performance.

**Figure 9:**
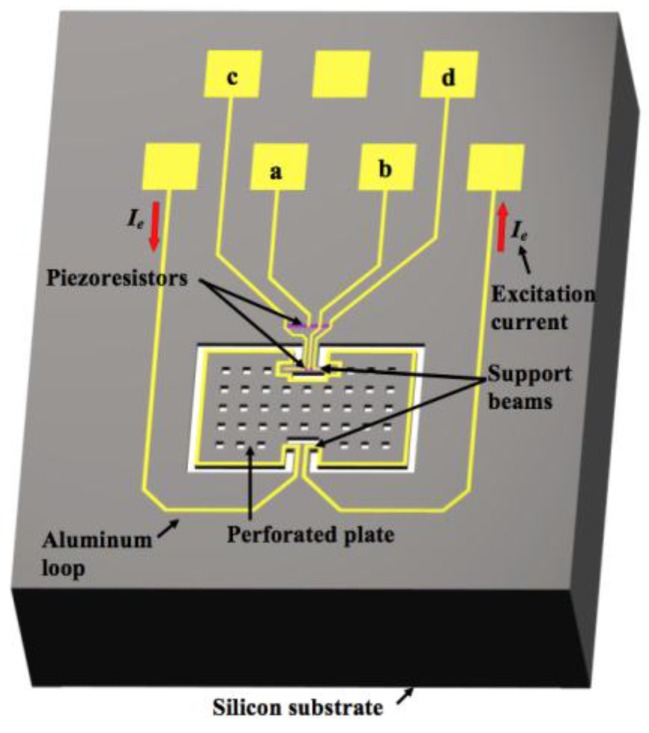
Schematic view of the main components of a MEMS magnetic field device designed by Herrera-May et al. [27]. Reprinted with permission from [27]. Copyright©2015, Elsevier B.V.

**Figure 10:**
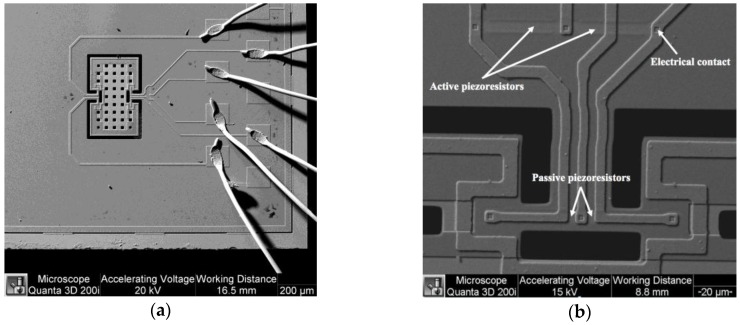
SEM image of a MEMS magnetic field device with piezoresistive sensing: (**a**) Silicon resonator and aluminum loop; (**b**) Wheatstone bridge with four piezoresistors [27]. Reprinted with permission from [27]. Copyright©2015, Elsevier B.V.

A MEMS magnetic field device (see Figure 9) with a simple resonator and linear electrical response was presented by Herrera-May et al. [27]. It is formed by a perforate plate (472 × 300 × 15 μm^3^), four flexural beams (18 × 15 × 15 μm^3^), two support beams (60 × 36 × 15 μm^3^) and a Wheatstone bridge with four p-type piezoresistors. The device exploits the Lorentz force and operates at its seesaw resonant frequency (100.7 kHz) without vacuum packaging. A standard bulk micromachining process and SOI wafers are used to fabricate the device, as shown in Figure 10. The dynamic range of the device can be adjusted by modifying the excitation electrical current, keeping its linear electrical response. It has a quality factor of 419.6, a sensitivity of 230 mV·T^−1^, a resolution of 2.5 μT and a power consumption of 12 mW. Due to the advantages of this sensor, it could be employed in applications of non-destructive magnetic testing to detect flaws and corrosion of ferromagnetic materials. For this application, the sensor needs reliability studies of its behavior under different conditions of temperature, moisture, and fatigue.

**Figure 11:**
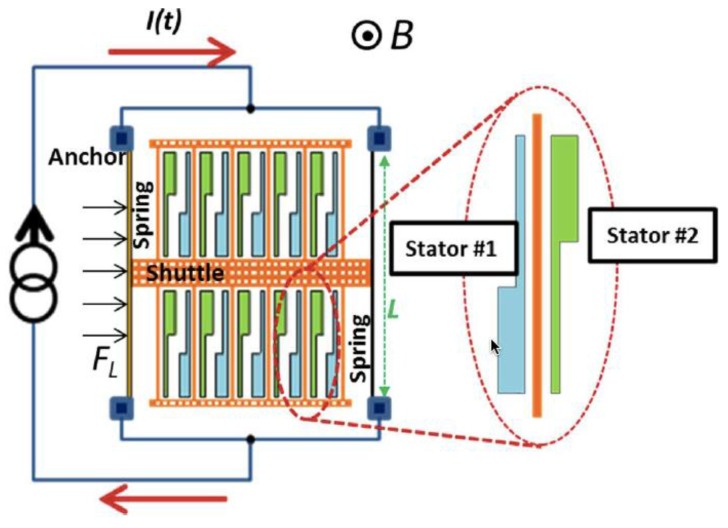
Schematic view of a MEMS magnetic field sensor formed with parallel plates, fixed stators, and a shuttle supported by two thin beams. This sensor uses the Lorentz force, which causes a displacement of its resonant structure measured through differential capacitors [28]. Reprinted with permission from [28]. Copyright©2013, IEEE.

**Figure 12:**
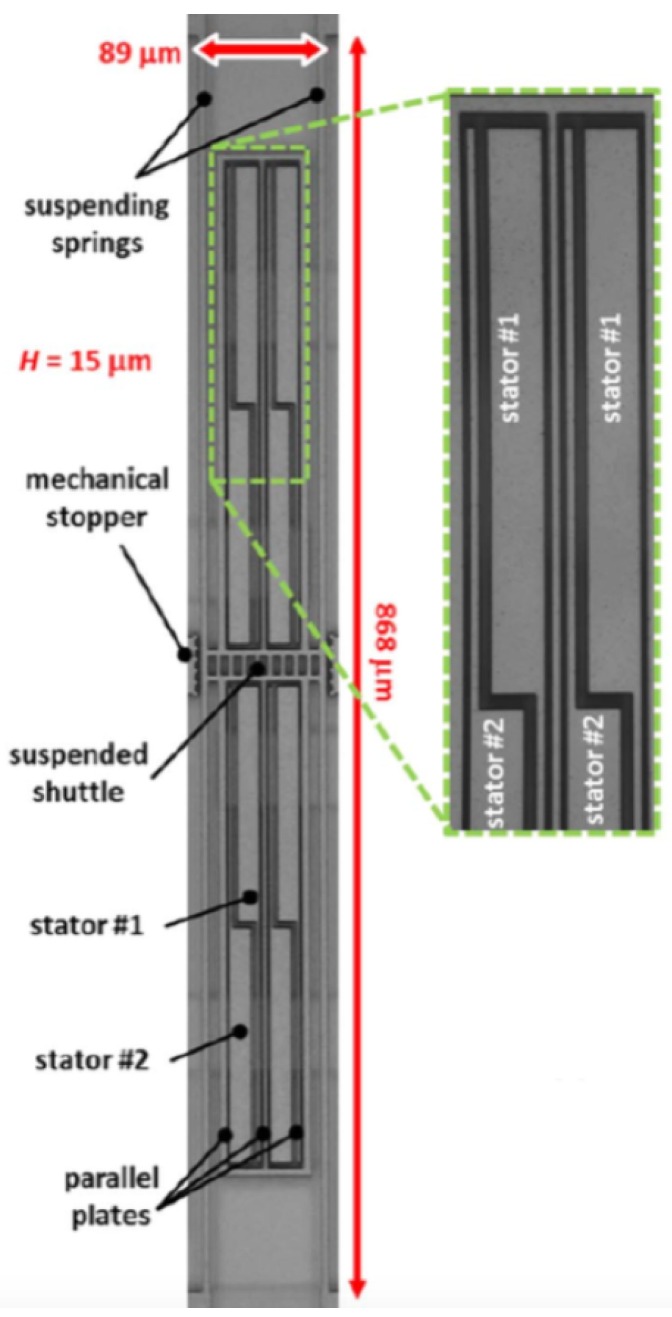
Microphotography of the main components of a MEMS magnetic field sensor with capacitive sensing [28]. Reprinted with permission from [28]. Copyright©2013, IEEE.

Figure 11 depicts a schematic view of a MEMS magnetic field sensor with capacitive readout technique [28]. This sensor detects magnetic field with orthogonal direction (*z*-axis along) to surface of the resonant structure. It consists of a set of fixed stators and a shuttle suspended with two thin beams (see Figure 11), which forms two differential parallel-plate sensing capacitors *C_1_* and *C_2_*. A Lorentz force is generated on two thin beams caused by the interaction between the magnetic field and ac electrical current flowing through the beams with the sensor resonant frequency. This force has an orthogonal direction to the plane of both magnetic field and ac current, causing a displacement of the beams and parallel plates. This displacement is detected through the differential capacitance variation between the parallel plates and fixed stators (see Figure 12). The sensor sensitivity is measured as the differential capacitance shift per variation of magnetic field. This sensor has an overall sensitivity of 150 μV·μT^−1^ at 250 μA of peak driving current, a theoretical noise of 557.2 μV·Hz^−1/2^, a resolution of 520 nT·mA^−1^·Hz^−1/2^, a quality factor around 328, a resonant frequency of 28.3 kHz and a typical industrial packaging. The technical characteristics of this sensor are suitable for consumer applications (e.g., digital compass, dead reckoning, heading, map rotation), although this sensor only detects the components of the magnetic field in one direction and its performance depends on the temperature. This sensor could include a temperature device, embedded on the same MEMS readout electronic, for post-acquisition compensation of temperature alterations. Therefore, this sensor requires initial calibration and temperature compensation.

**Figure 13:**
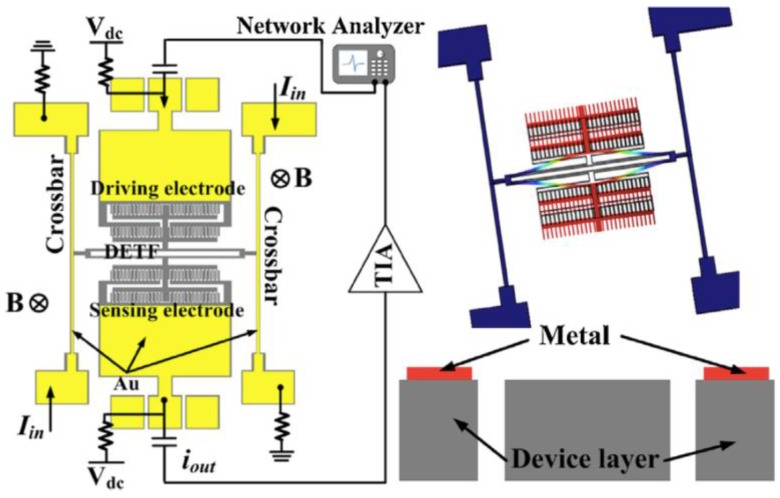
Schematic of a magnetic field sensor formed by a DETF silicon resonator, including the biasing configuration with a transimpedance amplifier (TIA) at the sensing port. The right side depicts the anti-phase vibration mode of the DETF resonator and a cross section schematic view of the sensor [29]. Reprinted with permission from [29]. Copyright©2014, Elsevier B.V.

**Figure 14:**
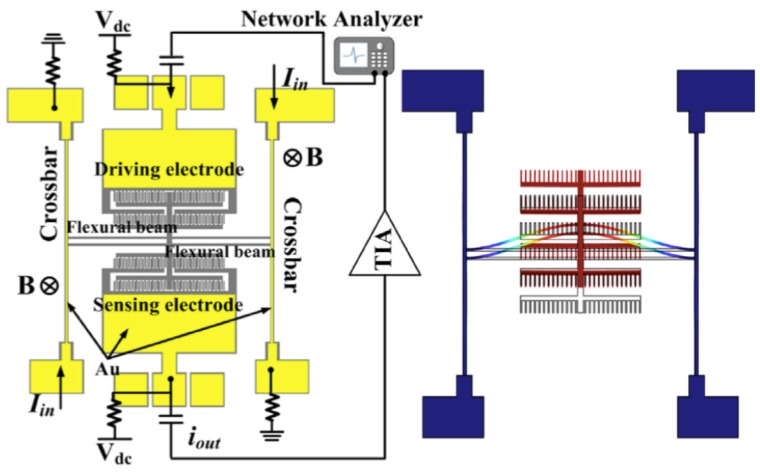
Schematic of a magnetic field sensor formed by a DETF silicon resonator, which considers the bias configuration with a transimpedance amplifier (TIA) at the sensing port. The right side shows the in-phase vibration mode of the DETF resonator [29]. Reprinted with permission from [29]. Copyright©2014, Elsevier B.V.

Zhang et al. [29] developed a silicon micromechanical magnetic field sensor based on a double-ended tuning fork (DETF) resonator, as shown in Figure 13. This silicon resonator has two parallel beams (600 × 8 μm^2^) clamped to two doubly fixed crossbars and two sets of comb drive electrodes joined to each parallel beam. The first set of comb drive electrodes operates as actuation mechanism and the second set of electrodes acts as a sensing mechanism. The DEFT resonator is driven using an electrostatic force that activates its in-plane (46.8 kHz) and anti-phase (49.3 kHz) vibration modes (Figure 13 and Figure 14). This sensor measures the resonant frequency shift using the Lorentz force. This force is generated by the interaction of an out-of-plane magnetic field and dc electrical current flowing through the two crossbars. The Lorentz force modifies the stiffness of the two parallel beams, which modifies the resonant frequency in function of the applied magnetic field. This sensor was fabricated using SOI wafers during a standard bulk micromachining process. Using a resonator of 10-thickness, the magnetic field sensor reaches sensitivities of 215.74 ppm/T and 203.71 ppm/T for the anti-phase and in-phase vibration mode, respectively. For anti-phase and in-phase vibration mode, the resonator has a quality factor of 100,000 and 42,000, respectively. However, the dc current supplied to the crossbars increases the crossbars temperature, causing a thermally-induced frequency shift. This effect is minimized by reducing the resistance across the crossbars through depositing a metal stack of chromium and gold along the crossbars. This sensor needs a vacuum packaging and more researches on the dependence of the sensor performance with respect to the Joule effect and damping sources.

**Figure 15:**
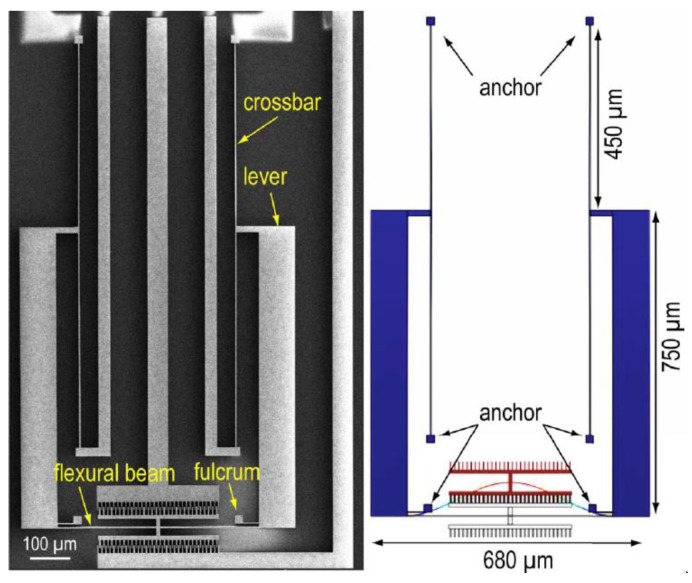
SEM image of magnetic field sensor (left) and its simulated vibration mode (right) [30]. Reprinted with permission from [30]. Copyright©2015, IEEE.

**Figure 16:**
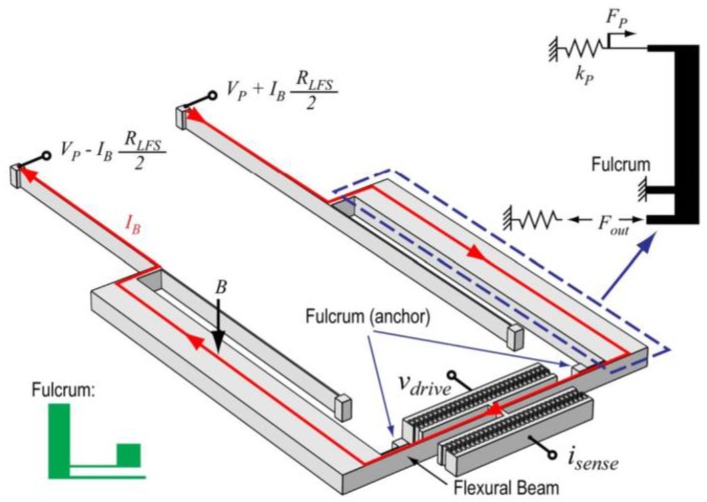
Operation principle of a magnetic field sensor with microleverage mechanism. A magnetic field (*B*) interacts with the DC excitation current (*I_B_*) to generate a Lorentz force, which operates as axial load on the flexural beam resonator. This force is mechanically amplified by the microleverage mechanism [30]. Reprinted with permission from [30]. Copyright©2015, IEEE.

Li et al. [30] designed a magnetic field sensor composed of a flexural beam resonator (1200 × 680 × 40 μm^3^), which is then coupled to current-carrying silicon beams through a microleverage mechanism. This resonator uses electrostatic actuation and capacitive sensing through 30 comb fingers on each side of the flexural beam, as shown in Figure 15. The sensor detects the magnetic field using the resonant frequency shift due to the Lorentz force, which operates as axial load on the resonator (Figure 16). The microleverage mechanism amplifies the tension produced by the Lorentz force, increasing the sensor’s sensitivity by a factor of 42 with respect to the same design without this mechanism. The sensor uses vacuum packaging obtained by using eutetic bonding between the two wafers. The sensor obtains a sensitivity of 6687 ppm/(mA·T), a noise floor of 0.5 ppm·Hz^−1/2^, a quality factor of 540 and a resonant frequency of 21.9 kHz. The sensor’s sensitivity improves when the quality factor increases. With this, the sensor could be used for compass applications. This sensor uses a large silicon beam (500 × 40 × 3 μm^3^) subject to flexural loads, which needs mechanical reliability testing. 

**Figure 17:**
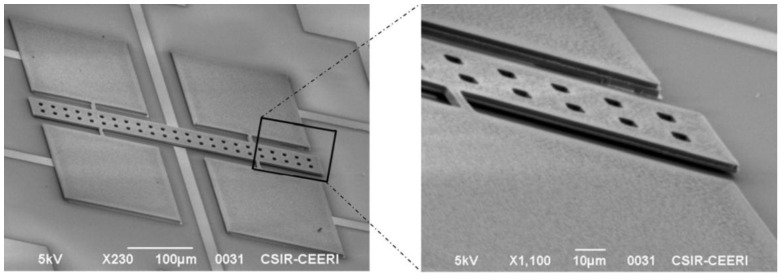
SEM image of a silicon xylophone supported by four beams, which take advantage of capacitive sensing to detect magnetic field [31]. Reprinted with permission from [31]. Copyright©2016, Springer International Publishing AG.

**Figure 18:**
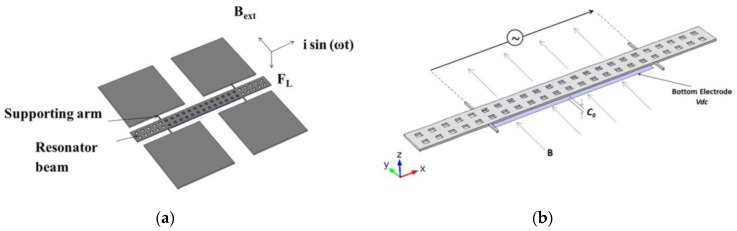
Schematic of the: (**a**) operation principle of a xylophone resonator; and (**b**) its electrical connections for capacitive sensing [31]. Reprinted with permission from [31]. Copyright©2016, Springer International Publishing AG.

Aditi and Gopal [31] fabricated a MEMS magnetic field sensor (see Figure 17) by anodic bonding technique using SOI and glass wafers. This sensor has a xylophone resonator of highly doped silicon without a metal top electrode. The device fabrication has the advantages following: a low temperature (≤400 °C) process, reliable, repeatable, reduced lithography steps and the ability to control the gap between the electrodes. In addition, the device uses a vacuum packaging (1200 Pa) at die level which is obtained through an anodic bonding process. The xylophone exploits the Lorentz force to operate its first resonant mode (108.75 kHz) with a quality factor of 180. The xylophone is electromagnetically actuated, causing displacements that are electrostatically sensed by capacitive method (see Figure 18). The device behaves as a parallel plate capacitor, in which a capacitive sensing circuit converts the capacitance shift to an electrical signal. It has a power consumption of 0.45 mW and a resolution of 215 nT·Hz^−1/2^. This sensor can be used for non-destructive magnetic testing of ferromagnetic materials. In addition, with an improvement in the vacuum pressure (about 10 Pa), this sensor could be employed as a compass in electronic devices and gadgets. For a constant magnetic field, the device registered a non-linear displacement variation when the input current magnitude overcomes the 400 μA.

**Figure 19:**
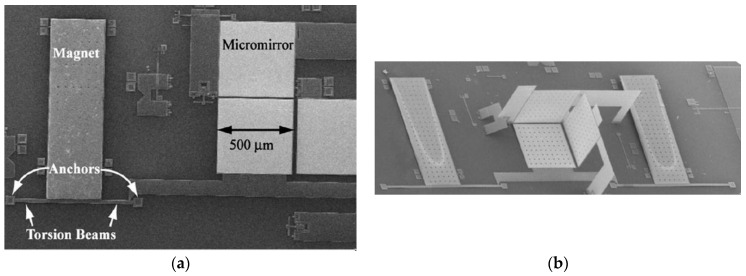
SEM image of a MEMS magnetic field sensor integrated with a CCR: (**a**) before; (**b**) after assembly [32]. Reprinted with permission from [32]. Copyright©2007, IEEE.

**Figure 20:**
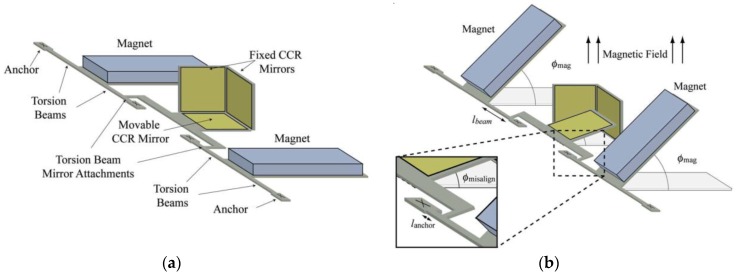
Schematic of a MEMS magnetic field sensor composed of a CCR: (**a**) without; (**b**) with an external magnetic field. In addition, a close up view of the coupling between the mirror and torsion beam is shown [32]. Reprinted with permission from [32]. Copyright©2007, IEEE.

Vasquez and Judy [32] designed a zero-power magnetic field sensor that is integrated with a micromachined corner-cube reflector (CCR), a commercially available diode laser and a photodetector array, as shown in Figure 19. The sensor is composed of a torsional polysilicon beam joined to a polysilicon plate and a permanent magnet (CoNi layer) is deposited on the plate. This magnet can rotate around the beam axis. On the other hand, the CCR is composed by an orthogonal array of two fixed polysilicon mirrors and a flexible mirror connected to the torsional beam. Thus, the flexible mirror is coupled to the magnet rotation, as shown in Figure 20. The CCR is fabricated using multi-user MEMS processes (MUMPs) [33]. When the magnetic field is zero, the three mirrors (500 × 500 μm^2^) of the CCR are perpendicular to one another. An alteration of the magnetic field produces a rotation of the magnet and flexible mirror, which is proportional to the magnetic field. Then, a diode laser is used to interrogate the CCR, splitting in two beams the reflected optical beam. The displacements of these optical beams are measured through a photodetector array. This sensor does not require power consumption and it can continuously operate in extremely harsh environments such as industrial, aerospace and the automotive sector. This sensor can detect a magnetic field from 1030 μT to 7540 μT with an uncertainty of 113 μT at 1-m optical-interrogation range. To improve the sensor’s performance the mirror curvature could be decreased and the mirror’s size and magnet volume increased, as well as attaching the mirrors directly to the magnet. This sensor has a potential application in wireless sensing systems that operate in harsh or hostile locations that do not need to provide sensor-node energy. However, more studies on the effects of etch holes on the sensor sensitivity should be performed.

**Figure 21:**
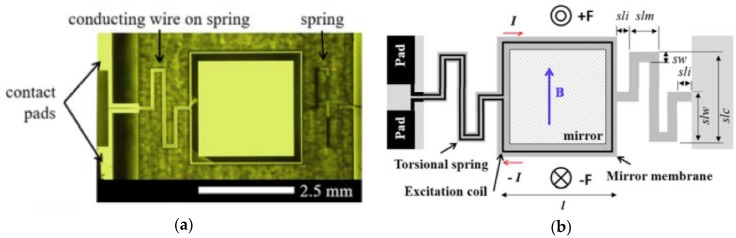
(**a**) Microscope image of a MEMS magnetic field sensor with optical readout; and (**b**) operation principle of the sensor [34]. Reprinted with permission from [34]. Copyright©2016, Elsevier B.V.

Park et al. [34] designed a magnetic field sensor formed by a silicon resonator and compact laser positioning system. This system has a photodetector and laser diode for monitoring the angular displacement of a current biased mirror membrane. The resonator is composed of a silicon membrane (3000 × 3000 × 12 μm^3^) coated with an aluminum layer (2500 × 2500 × 0.8 μm^3^). The membrane is supported by two torsional springs (2100 × 100 × 12 μm^3^), in which an aluminum wire (30 μm width and 0.8 μm thickness) is deposited, as shown in Figure 21. The sensor exploits the Lorentz force in order to generate a rotational motion of the mirror that is measured with the laser positioning system. To increase the sensitivity of the sensor, the mirror oscillates at resonance (torsional vibration mode) at atmospheric pressure. Thus, the applied magnetic field is related to the displacements of the mirror. For a coil bias current of 50 mA at atmospheric pressure, the sensor registers a sensitivity of 62 mV·μT^−1^, a resonant frequency of 364 Hz, a quality factor of 116, a resolution of 0.4 nT with a bandwidth of 53 mHz and noise floor level of 1.78 nT·Hz^−1/2^. This sensor can detect a magnetic field between a range of nanoTeslas and Teslas. Nevertheless, the variation of the sensor behavior due to heating of the membrane and springs should be studied.

## 3. Potential Applications

**Figure 22:**
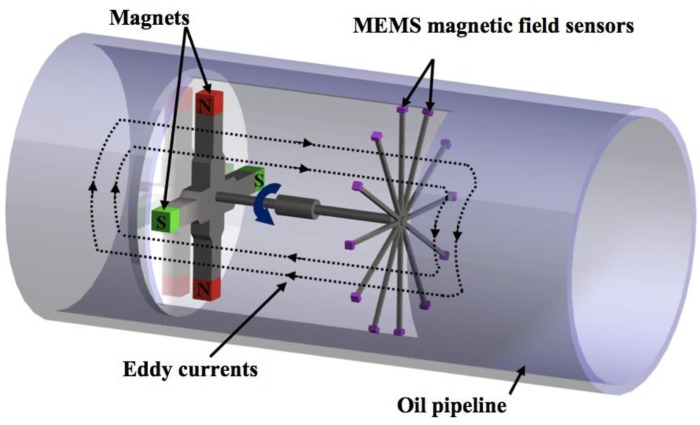
Design of a flaws inspection system in oil pipeline using Eddy current technique [36]. Reprinted with permission from [36]. Copyright©2011, InTech.

**Figure 23:**
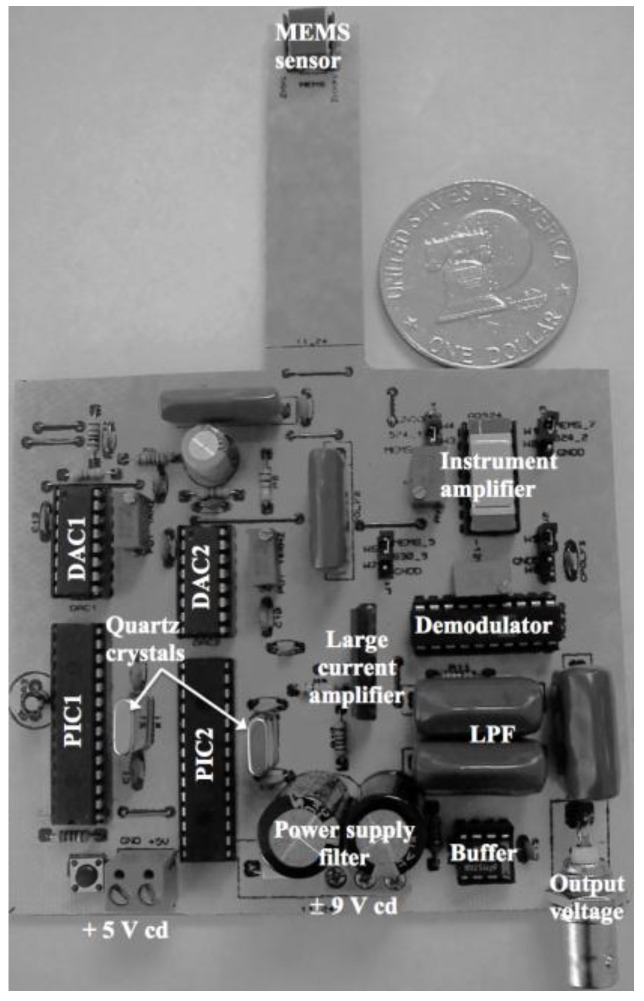
Printed circuit board (PCB) for a portable signal conditioning system of a MEMS sensor, which could be used for monitoring residual magnetic field of ferromagnetic materials [37]. Reprinted with permission from [37]. Copyright©2016, Springer International Publishing AG.

Magnetic field sensors based on MEMS resonators have potential applications such as telecommunications, military, industrial, automotive, biomedical and consumer electronic products. This is due to the important advantages of MEMS sensors, which include small size, lightweight, low power consumer, wide dynamic range, compact signal conditioning and high sensitivity. These sensors could detect flaws or residual stresses of ferromagnetic materials using non-destructive testing such as Eddy current inspection and the magnetic memory method (MMM) [35,36,37]. Eddy currents are generated on the surface of the ferromagnetic material when a variable magnetic field interacts with this surface. The flaws of the ferromagnetic material alter the Eddy currents, modifying their magnetic field in relation to the size of the flaws. For instance, an array of magnetic field sensors could detect flaws of an oil pipeline (see Figure 22) using the Eddy currents technique [36]. On the other hand, MMM can detect flaws or residual stress of ferromagnetic materials through the variations of their residual magnetic field. Thus, magnetic field sensors could measure these modifications of a magnetic field related to the size of the flaws, or magnitude of the residual stress. Lara-Castro et al. [37] developed a portable signal conditioning system of a magnetic field sensor (see Figure 23), which could detect residual magnetic field of ferromagnetic materials using the MMM. 

**Figure 24:**
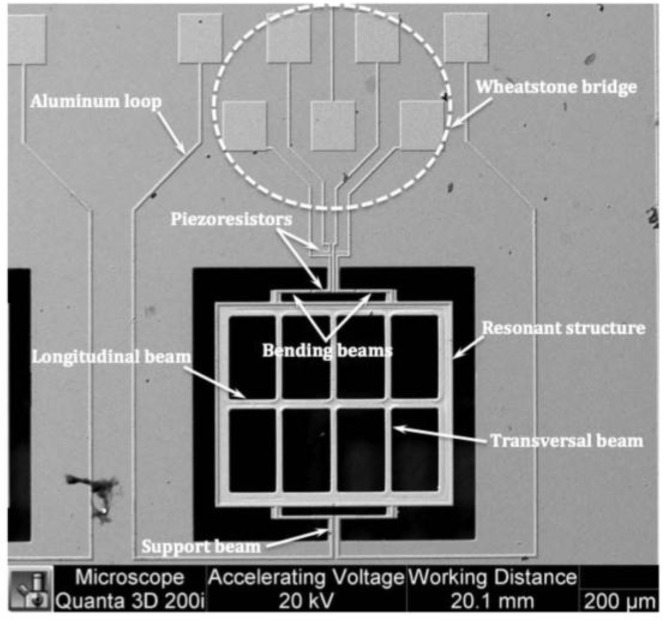
SEM image of a MEMS magnetic field sensor with potential application to obtain a respiratory magnetogram [38]. Reprinted with permission from [38]. Copyright©2013, Ivyspring International Publisher.

**Figure 25:**
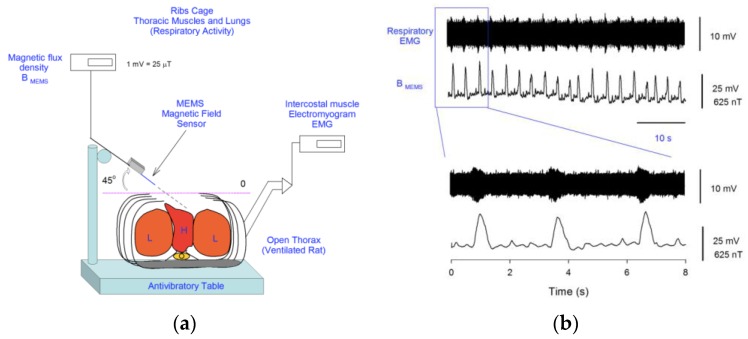
(**a**) Diagram of an experimental set up of a MEMS magnetic field sensor for monitoring the respiratory and cardiac activity of a rat; (**b**) Electromyogram of the thoracic muscles and magnetogram of the thoracic cavity during the respiratory activity of a rat [38]. Reprinted with permission from [38]. Copyright©2013, Ivyspring International Publisher.

Domínguez-Nicolas et al. [38] developed a respiratory magnetogram employing a MEMS magnetic field sensor with piezoresistive sensing and silicon resonator (see Figure 24). This device measured the magnetic field during the respiratory activity of rats. These researchers registered the magnetogram and electromyogram of the thoracic cavity of a rat during its respiration, as shown in Figure 25. For this magnetogram, Juarez-Aguirre et al. [39] designed a digital signal processing through virtual instrumentation. A future application of this magnetogram could be used for monitoring the health of some organs of the thoracic cavity. For instance, healthy organs of the thoracic cavity (e.g., heart) could generate magnetic fields in a determined range; unhealthy organs however, could cause abnormal magnetic fields. More research related to magnetic fields emitted by healthy and unhealthy organs of the thoracic cavity is required. In addition, Tapia et al. [40] built an electronic neuron (FitzHugh-Nagumo) to generate controlled spike-like magnetic fields, which were measured with a MEMS sensor. In future applications, this sensor could be improved to detect spiking activity of neurons or muscle cells. 

**Figure 26:**
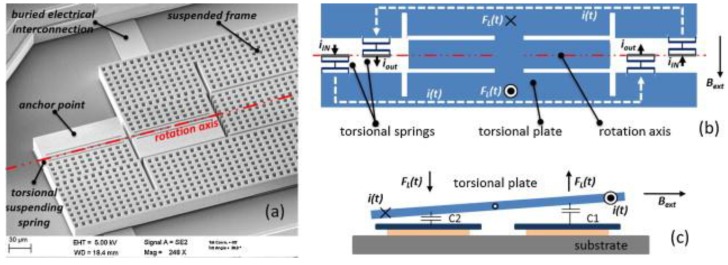
(**a**) SEM image of a MEMS magnetic field sensor with capacitive sensing for IMUs applications; (**b**) Top view; and (**c**) cross-section of the operating principle of the sensor [46]. Reprinted with permission from [46]. Copyright©2015, Elsevier B. V.

Other future applications of MEMS sensors include the micro-, nano- and pico-satellites. Miniaturization of satellites allows for the reduction of their launch costs, which can be achieved using sensors of small size, reliable, low energy consumption and high sensitivity. For instance, satellites must measure small magnetic fields during their space missions. For this application, Lamy et al. [41] and Ranvier et al. [42] designed MEMS sensors composed of polysilicon-xylophone bars and a capacitive sensing. In addition, space satellites need inertial measurement units (IMUs) formed by magnetic field sensors, accelerometers and gyroscopes. These IMUs could be fabricated on a single chip to decrease the power consumption and electronic noise. Other IMUs applications will include the navigation systems of ships, trains, military and civil aviation and unmanned operated vehicles [43,44,45]. Laghi et al. [46] fabricated a torsional MEMS sensor through surface micromachining for monitoring in-plane magnetic field, as shown in Figure 26. This sensor operates with the Lorentz force and capacitive sensing. This device has the following technical parameters: size of 282 × 1095 μm^2^, vacuum packaging of 0.35 mbar, resonant frequency of 19.95 kHz, quality factor of 2500, a sensitivity of 0.85 V·mT^−1^ and a detection limit of 120 nT·mA·Hz^−1/2^.

**Figure 27:**
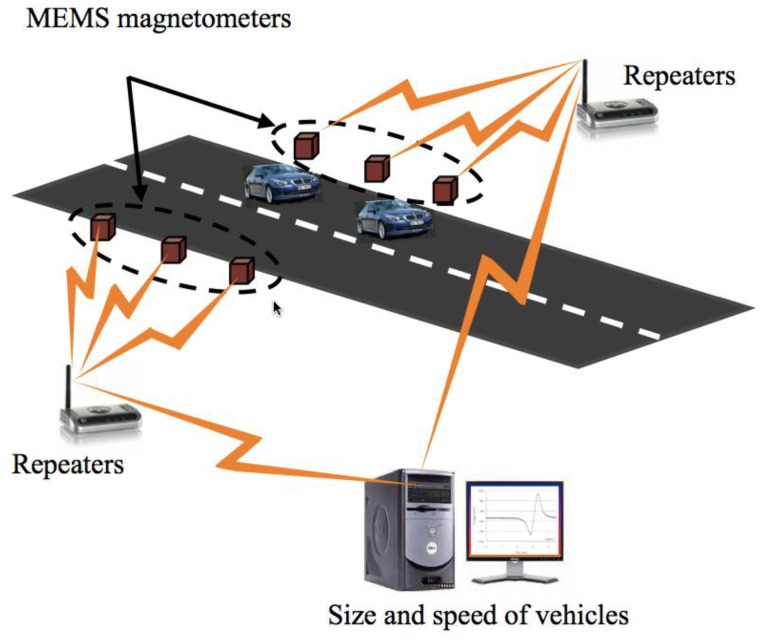
Schematic view of traffic detection system formed by MEMS devices and signal processing [36]. Reprinted with permission from [36]. Copyright@2011, Intech.

Traffic detection systems could measure the speed and size of vehicles considering MEMS devices, which are located in parallel alongside the road [36]. These devices will have a constant separation distance and will detect the variation of Earth’s magnetic field generated by the motion of the vehicles, as shown in Figure 27. These changes can be measured at different times (*t*_1_ and *t*_2_) using the MEMS devices. Thus, vehicle speed will be determined using the ratio of the devices separation distance to the time difference *t*_1_ and *t*_2_. The vehicle size will be related with the magnitude change of the Earth’s magnetic field.

## 4. Comparisons and Challenges

MEMS resonators used in Lorentz force-based magnetic field sensors offer several advantages with respect to conventional sensors, allowing for their use in future applications. The Lorentz force-based sensors provide a wide measurement range by changing the magnitude of the excitation current. In addition, vacuum packaging reduces the air damping, increasing the quality factor and sensitivity of the MEMS resonators. These sensors need a low energy consumption and have a compact structure, which can be fabricated through a standard micromachining process. Also, MEMS sensors can use different sensing techniques (e.g., piezoresistive, optical, or capacitive) to detect magnetic field. Each technique presents specific benefits that are employed for the designers to develop the best sensor for a specific application. For example, piezoresistive sensing is adequate for the bulk micromachining process and simple signal processing systems. With these systems, an electrical output signal related with the magnetic field is obtained. The piezoresistive sensing registers a voltage offset and requires temperature compensation circuits. On the other hand, capacitive sensing is mostly used in the superficial micromachining process and it converts the applied magnetic field into an electrical output signal. This technique has little temperature dependence and allows the fabrication of electronic circuits on the same chip of the magnetic field sensor. It permits the reduction of the device size and parasitic capacitances. Generally, the sensors with capacitive sensing have high air damping at atmospheric pressure; therefore they need vacuum packaging to increase their sensitivity. Finally, optical sensing has immunity to electromagnetic interference (EMI) and demands less electronic circuitry than both capacitive and piezoresistive sensing. Sensors with optical sensing can operate in hostile or harsh environments without providing energy for the sensor. The superficial and bulk micromachining processes are suitable for this sensing technique. Nevertheless, all these sensing techniques have problems with the heating of the sensor structure due to the Joule effect, generating thermal stress and displacements of the resonators. More studies about the modifications in the behavior of the MEMS resonators due to the Joule effect must be performed. In addition, the mechanical reliability of the resonators must be studied to ensure the best performance of the MEMS sensors.

**Figure 28:**
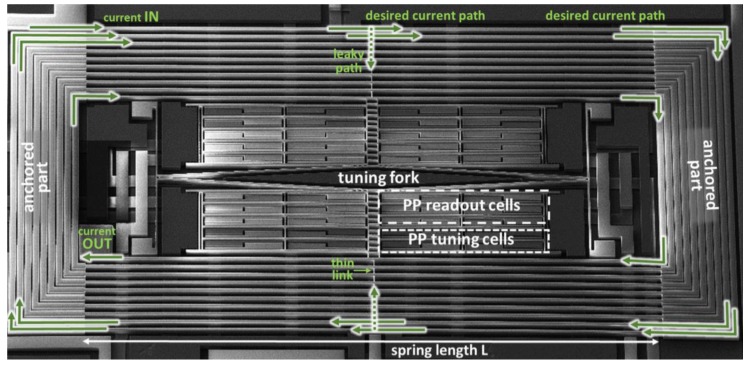
SEM image of diamond-shaped tuning fork, including 10 metal coils deposited over the springs and parallel plate (PP) cells. These cells are employed for capacitive readout and tuning [47]. Reprinted with permission from [47]. Copyright@2015, IEEE.

**Figure 29:**
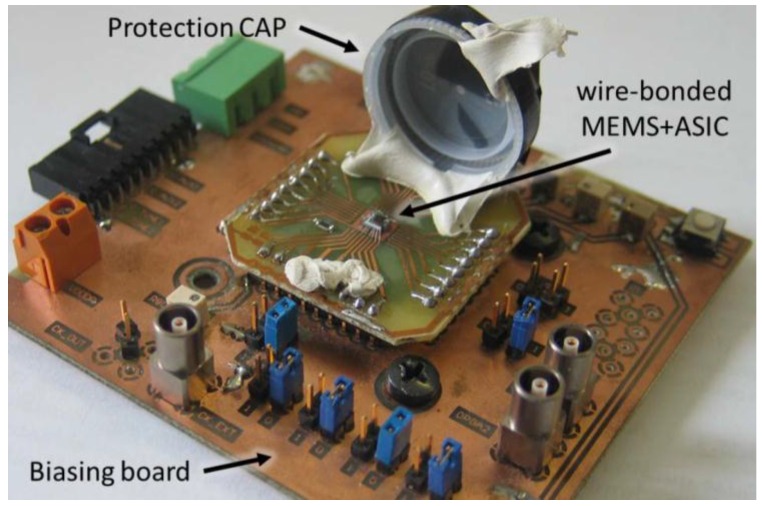
Photography showing the staked MEMS and ASIC dies, which are wire-bonded on a socket carrier located on the biasing PCB board [47]. Reprinted with permission from [47]. Copyright@2015, IEEE.

Table 1 depicts some characteristics of magnetic field sensors composed by resonators and fabricated with micromachining process. Recently, MEMS magnetic field devices have been developed with important advantages for future commercial markets. To guarantee a reliable performance of these devices, more researches must be made with respect to vacuum packaging, dependence on changes of humidity and temperature, as well as a reduction of voltage offset and noise. With respect to the reduction of electronic noise, Minotti et al. [47] developed a magnetic field sensor (see Figure 28) with a custom integrated readout circuit containing a capacitive-sensing front-end, a mixer and a low-pass filter for signal demodulation. This sensor has tuning fork geometry to reject accelerations and vibrations and multiple loops to increase its sensitivity. It operates in off-resonance mode to overcome both trade-offs between bandwidth and resolution and long-term stability. The sensor is fabricated using the thick epitaxial layer for microactuators and accelerometers (ThELMA) process from STMicroelectronics [28]. A 0.35-μm CMOS process from AustriaMicroSystem (AMS) is used to fabricate the integrated circuit. In addition, the overall system (see Figure 29) has a low consumption of power close to 775 μW, a sensitivity of 0.75 zF·nT^−1^·mA^−1^, and packaging pressures of about 0.75 mbar. Thus, it can reach large full-scale ranges up to ±2.4 mT, adjusting the driving current, which exceeds the full-scale of conventional devices such as the Hall effect and anisotropic magneto-resistance (AMR) [48].

Reliability studies are required to know the devices behavior under different environments and operation conditions. In addition, studies of the main damping sources of resonators must be considered in the design phase of devices [49,50]. Designers can use these studies to obtain resonators with minimum damping, which will improve their quality factor and resolution. Vacuum packaging can decrease the air damping at resonators, increasing their performance. Future applications need multifunctional sensors for monitoring several physical parameters (e.g., acceleration, magnetic field, angular ratio, humidity, temperature and gases). For this, MEMS technology will allow the integration of different sensors on a single chip. Future energy sources as microgenerators incorporated into the devices could provide them with their own power, therefore eliminating batteries. Also, more studies of magnetic stochastic resonance could be made to improve the detection of magnetic fields. This stochastic resonance applied to magnetic field sensors could help to increase their dynamic range and sensitivity [51]. For this reason, sensors should consider stochastic-resonance coils on the same chip, in which these coils could be fabricated with metallic coils around the active area of the sensors.

## 5. Conclusions

Magnetic field sensors formed by MEMS resonators have important advantages in respect to conventional devices, allowing for their implementation in future applications. Most of these sensors use silicon resonators that exploit the Lorentz force and different sensing techniques such as piezoresistive, optical, or capacitive. The piezoresistive sensing is a simple technique with an easy fabrication process; although it can have voltage offset and temperature dependence. Capacitive sensing has little temperature dependence but suffers from parasitic capacitances, which can be decreased with monolithic fabrication. Optical readout offers immunity to electromagnetic interference (EMI) and decreases the electronic components. The main fabrication processes include surface and bulk micromachining, considering materials such as silicon, polysilicon, silicon dioxide, aluminum and gold. However, MEMS devices need more reliability research in order to predict their performance under different environments and operation conditions. Future works must consider the reduction of damping and electronic noise, as well as the integration of different sensors on a single chip for monitoring several physical parameters.

## Figures and Tables

**Table 1:** Main characteristics of recent MEMS magnetic field sensors based on Lorentz force.

Magnetic Field Sensor	Resonator Size (μm × μm)	Resonant Frequency (kHz)	Quality Factor	Noise	Detection Limit	Sensitivity
Herrera-May et al. [25]	400 × 150	136.52	842	57.5 nV∙Hz^−1/2^ *	143 nT∙Hz^−1/2^ *	403 mV∙T^−1^
Mehdizadeh et al. [26]	500 × 1400	2550	16,900	--- **	---^**^	262 mV∙T^−1^
Herrera-May et al. [27]	472 × 300	100.7	419.6	37.1 nV∙Hz^−1/2^ *	161 nT∙Hz^−1/2^ *	230 mV∙T^−1^
Zhang et al. [29]	600 × 800	49.3	100,000	--- **	--- **	215.74 ppm∙T^−1^
Langfelder et al. [28]	89 × 868	28.3	327.9	557.2 μV∙Hz^−1/2^ *	520 nT∙mA∙Hz^−1/2^	150 μV∙μT^−1^
Li et al. [30]	1200 × 680	21.9	540	0.5 ppm∙Hz^−1/2^	--- **	6687 ppm∙mA^−1^∙T^−1^
Laghi et al. [46]	282 × 1095	19.95	2,500	--- **	120 nT∙mA∙Hz^−1/2^	0.85 V∙mT^−1^
Minotti et al. [47]	1700 × 750	20	460	30 zF∙Hz^−1/2^	40 nT∙mA∙Hz^−1/2^	0.75 zF∙nT^−1^∙mA^−1^
Park et al. [34]	3000 × 3000	0.36	116	1.78 nT∙Hz^−1/2^	0.4 nT with BW = 53 mHz	62 mV∙μT^−1^

* Theoretical data; ** Data do not available in the literature.
